# Image-based RNA interference screening reveals an individual dependence of acute lymphoblastic leukemia on stromal cysteine support

**DOI:** 10.18632/oncotarget.2572

**Published:** 2014-11-08

**Authors:** Jeannette Boutter, Yun Huang, Blerim Marovca, Andreas Vonderheit, Michael A. Grotzer, Cornelia Eckert, Gunnar Cario, Bernd Wollscheid, Peter Horvath, Beat C. Bornhauser, Jean-Pierre Bourquin

**Affiliations:** ^1^ Department of Pediatric Oncology, University Children's Hospital Zurich, Zurich, Switzerland; ^2^ Institute of Molecular Biology, Mainz, Germany; ^3^ Department of Pediatric Oncology/Hematology, Charité Universitätsmedizin Berlin, Germany; ^4^ Department of Pediatrics, University Medical Centre Schleswig-Holstein, Kiel, Germany; ^5^ Department of Biology, Institute of Molecular Systems Biology, ETH Zurich, Zurich, Switzerland; ^6^ Synthetic and Systems Biology Unit, Biological Research Center, Szeged, Hungary; ^7^ Institute for Molecular Medicine Finland (FIMM), University of Helsinki, Helsinki, Finland; ^8^ Children's Research Center (CRC), University Children's Hospital Zurich, Zurich, Switzerland

**Keywords:** acute lymphoblastic leukemia, bone marrow stroma, microenvironment, oxidative stress, RNAi screen

## Abstract

Interactions with the bone marrow microenvironment are essential for leukemia survival and disease progression. We developed an imaging-based RNAi platform to identify protective cues from bone marrow derived mesenchymal stromal cells (MSC) that promote survival of primary acute lymphoblastic leukemia (ALL) cells. Using a candidate gene approach, we detected distinct responses of individual ALL cases to RNA interference with stromal targets. The strongest effects were observed when interfering with solute carrier family 3 member 2 (SLC3A2) expression, which forms the cystine transporter x_c_^−^ when associated with SLC7A11. Import of cystine and metabolism to cysteine by stromal cells provides the limiting substrate to generate and maintain glutathione in ALL. This metabolic interaction reduces oxidative stress in ALL cells that depend on stromal x_c_^−^. Indeed, cysteine depletion using cysteine dioxygenase resulted in leukemia cell death. Thus, functional evaluation of intercellular interactions between leukemia cells and their microenvironment identifies a selective dependency of ALL cells on stromal metabolism for a relevant subgroup of cases, providing new opportunities to develop more personalized approaches to leukemia treatment.

## INTRODUCTION

Acute lymphoblastic leukemia (ALL) is the most common malignancy in childhood. The treatment of ALL is very effective, but the management of relapsed or refractory disease remains challenging [1]. Leukemia cells subvert normal hematopoietic stem cell niches, which provide protective cues to promote leukemia survival and disease propagation [2]. The intercellular crosstalk between leukemia cells and their microenvironment influences the biology of the disease and could contribute to drug resistance *in vivo* [3–4]. The cellular composition of the leukemia niche and the nature of the protective mechanisms between niche components remain poorly understood. The discovery of leukemia-specific interaction patterns between leukemia and niche cells will provide new targets to fight disease recurrence and improve the therapeutic window between leukemia cells and their normal counterpart.

Important components of the hematopoietic niche derive from skeletal stem cells in the bone marrow [5–7]. Evidence from mouse models indicates that these mesenchymal stem cells support normal hematopoiesis in the perivascular space [7–8], and the existence of specific niches for cells in distinct states of hematopoietic diffentiation was suggested [8]. Indeed, normal hematopoietic stem cells were shown to reside in a perivascular niche, while more mature lymphoid progenitors were found at endosteal niches [9–10]. Some molecular factors that contribute as niche factors to normal hematopoietic stem cell physiology have recently been identified [8]. In contrast, the molecular mechanisms that contribute specifically to ALL survival have remained elusive so far. *In vitro* models have employed explanted human bone marrow mesenchymal stromal cells (MSC) to support long term cultures of ALL cells [11–12]. These MSC are able to reconstitute functional hematopoietic niches *in vivo* after xenotransplantation [13–14].

Taking advantage of the leukemia-supporting function of MSC in vitro, we developed an image-based RNAi screening platform for functional genomic interrogation of the intercellular crosstalk between leukemia and bone marrow derived MSC. Our recently established xenograft model of ALL enabled systematic functional evaluation of patient derived primary leukemia samples [15–16]. Using a candidate gene approach, we identified patient specific patterns of dependence on stromal gene expression, involving multiple pathways. The most detrimental effect on leukemia survival was achieved by down-regulation of stromal solute carrier family 3 member 2 (SLC3A2), a subunit of the cystine transporter x_c_^−^. The survival of a subset of ALL samples was critically dependent on metabolic support from stromal cells, mediated by this amino acid transporter. We demonstrate that this protective mechanism involved stromal production of cysteine to maintain glutathione levels in leukemia cells, which results in protection from oxidative stress. Our approach detects relevant individual differences in functional interactions between leukemia and stromal cells, which has important implications for preclinical research.

## RESULTS

### Bone marrow mesenchymal stromal cells support primary ALL viability through heterogeneous mechanisms

**Figure 1:**
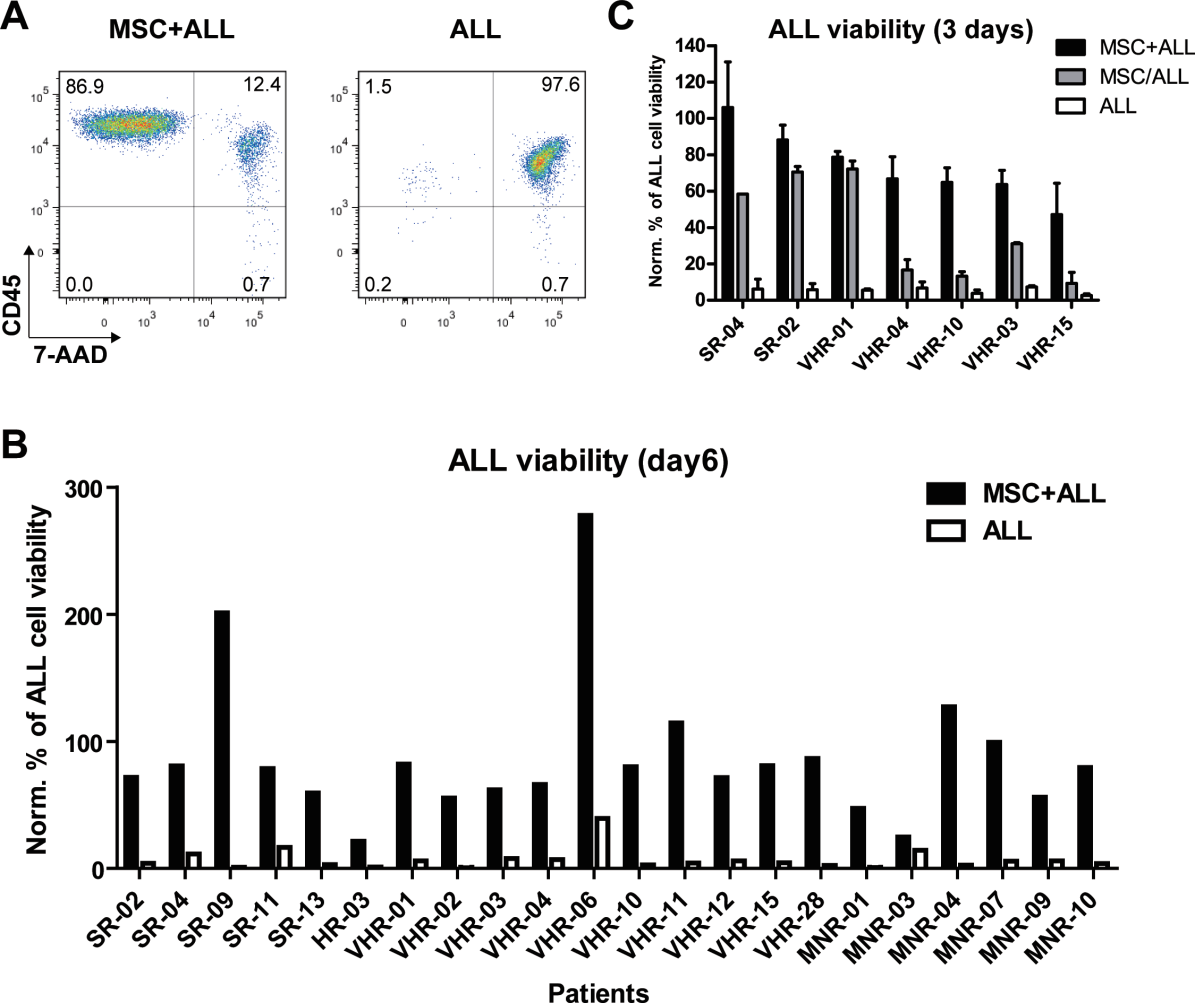
Bone marrow derived mesenchymal stromal cells (MSC) provide different pro-survival cues to support precursor B-cell ALL **(A)** ALL cell viability in monoculture or in co-culture with MSC after 6 days was measured by flow cytometry using anti-CD45 and 7-AAD stainings. **(B)** 22 BCP-ALL samples were tested for their survival in co-culture with MSC or in mono-culture after 6 days. The percentage of viable cells at day 6 was normalized to the input viable cell numbers. SR, standard risk; MR, medium risk; HR, high risk; VHR, very high risk; MNR, morphological non-responder, relapsed ALL cases. **(C)** Comparison of ALL cell viability in co-culture with MSC (MSC+ALL), separated via a transwell (MSC/ALL) or in mono-culture (ALL) after 3 days. Viability was assessed by FACS and performed at least in duplicates and normalized to the viable cell numbers of input.

Bone marrow derived, hTERT-immortalized MSC have been established to support leukemia cell lines survival in a model of the leukemia microenvironment [12]. With the aim to test whether this system is suitable for functional investigation of critical interactions between primary leukemia and stromal cells, we monitored the survival kinetics of 22 BCP ALL samples on human MSC (Fig. 1 and Supplemental Table S1). These samples included cases from different prognostic groups based on clinical criteria [17] (Supplemental Table S1). In monocultures, the viability of ALL cells dramatically decreased within 6 days (average residual viability = 6.99% of input) (Fig. 1A and B). A marked improvement of ALL cell survival (>50% of input cells after 6 days) was observed in co-cultures with MSC in serum free medium in 19 out of 22 ALL samples (Fig. 1A and B). In two cases a marked increase of cell number was observed. To evaluate the relative contribution of direct cell to cell contact for ALL support by MSC we used a transwell system to physically separate the two compartments. In two cases sufficient support was provided by the soluble fraction, while five cases depended largely on a direct cell-cell contact (Fig. 1C). Thus, distinct signals are required to support individual ALL cases. Based on these observations, we hypothesized that this ALL co-culture system could serve as a model to identify relevant interactions between leukemia cells and their microenvironment.

### A live cell imaging-based RNA interference platform to identify determinants from human MSC that support leukemia cell viability

**Figure 2:**
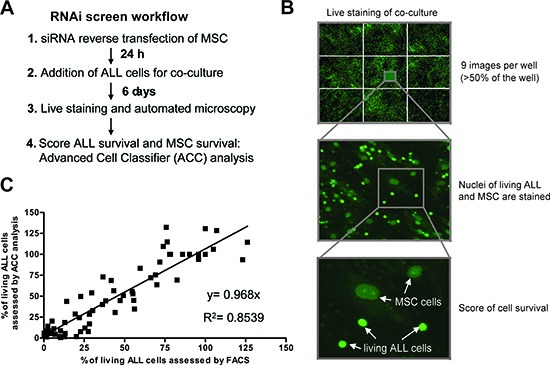
Live cell imaging based RNAi screening platform to examine ALL cell viability in co-cultures **(A)** Workflow of imaging-based assessment of ALL cell viability after RNA interference in MSC. **(B)** Living cells in the co-culture were stained using CyQUANT in a 384 well format. After automated microscopy which acquired 9 images per well covering more than 50% of each well and subsequent image segmentation using CellProfiler, the number of living ALL cells was assessed with the machine learning software Advanced Cell Classifier (ACC). **(C)** Correlation of the viability assessment of ALL cells by ACC and by flow cytometry. In total, 5 experiments were run in parallel for both methods. To get the full range of possible ALL cell viabilities, conditions with negative control siRNAs and positive control siRNAs (killing the MSC) as well as incubation with chemotherapeutic agents (dexamethasone, daunorubicin and vincristine) in different concentrations and seeding different numbers of cells were used.

We established a workflow for RNA interference in MSC cells (Fig. 2A) and an image-based screening platform to monitor ALL cells viability in a 384 well format (Fig. 2B). The best discrimination between living ALL and MSC cells was obtained using a live cell fluorescent dye (CyQUANT) with a modified staining protocol. The two cellular compartments can be analysed simultaneously in the same fluorescence channel based on the size, intensity and structure of the nuclei (Fig. 2B). An ‘a trous’ wavelet transform-based spot detection algorithm was implemented in the CellProfiler software to identify ALL cells [18–19]. To further avoid misdetections, ALL cells were classified using the machine-learning program Advanced Cell Classifier (ACC) [20]. A good correlation of the cell viability results obtained by automated microscopy and by flow cytometry using 7-AAD staining of the same experiments was reproducibly achieved (Fig. 2C). This provides the basis for large scale functional analysis of a two component cell culture system via high-content imaging and multi-parametric analysis using primary human cells.

### Heterogeneous requirement of MSC derived pro-survival cues for individual leukemias

**Figure 3:**
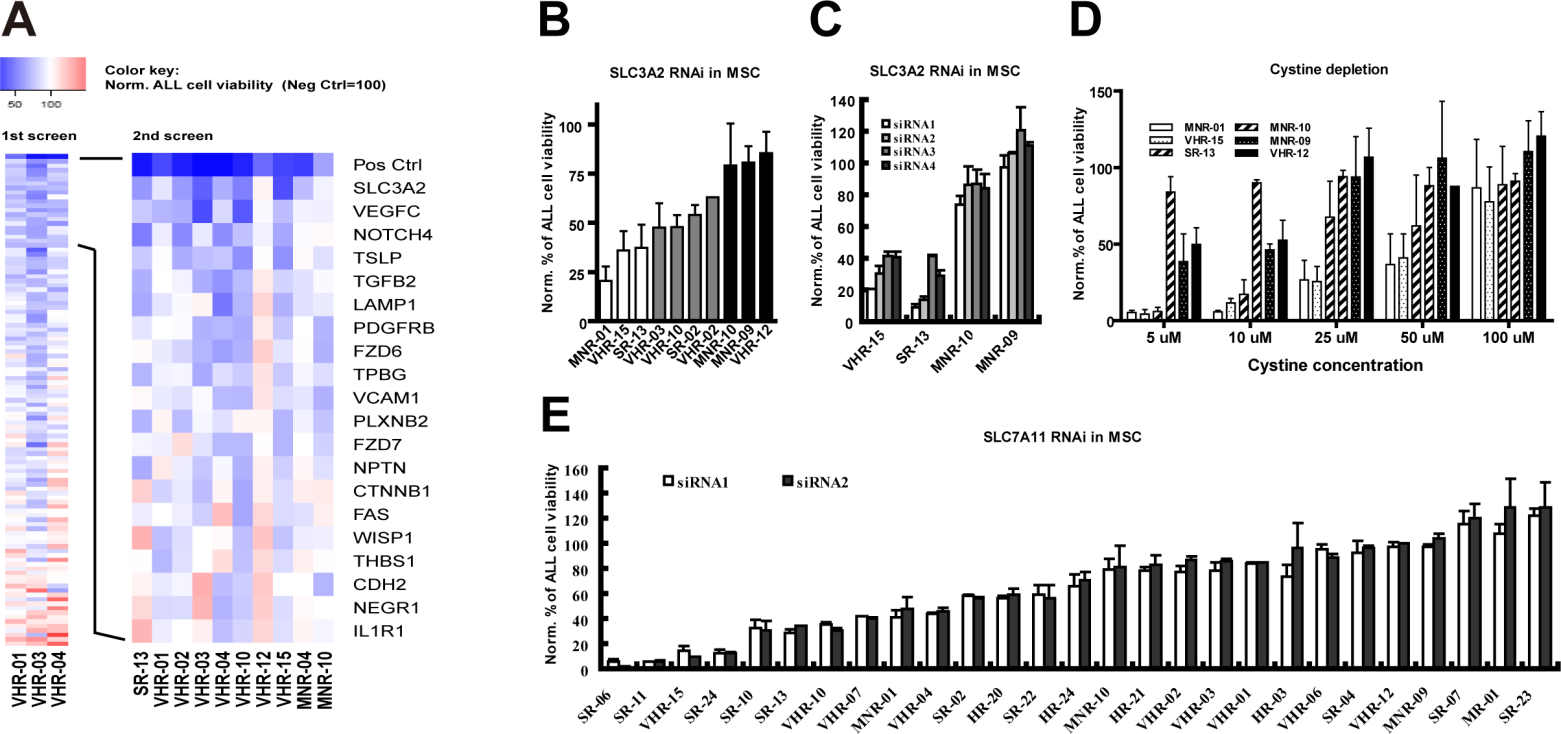
The stromal cystine transporter x_c_^−^ is essential for maintaining the viability of a subset of ALL cases **(A)** Identification of patient specific signals from MSC supporting ALL cell viability by RNAi interference. In the first screen, the effect of RNA interference with 110 genes in MSC on the viability of three ALL cases was assessed. 20 genes were found to significantly decrease ALL cell viability after RNA interference in MSC in at least 2 of the three cases. In the second screen, the effect of RNA interference with these 20 genes in MSC was assessed for 10 ALL cases. Depicted is the mean survival for each patient sample, normalized to the mean survival in the scrambled controls using the following color code: blue, decrease; red, increase. Neg Ctrl, scramble siRNA; Pos Ctrl, toxic siRNA that kills MSC. **(B)** ALL cell viability was assessed by flow cytometry for 10 patient samples after RNA interference with SLC3A2 at MSC level. The 3 most sensitive patients (white bars) and the 3 most resistant patients (black bars) were used for further experiments. **(C)** ALL cell viability was assessed by flow cytometry for 2 SLC3A2-dependent and 2 SLC3A2-independent cases after RNA interference with SLC3A2 in MSC using 4 individual siRNA sequences. **(D)** Cell viability assessed by flow cytometry after 6 days of co-culture with MSC in cystine depleted RPMI medium was decreased in 3 SLC3A2-dependent compared to 3 SLC3A2-independent samples. The supplementation of cystine rescued cell viability in a dose dependent manner. **(E)** Cell viability assessed by flow cytometry for 27 precursor B-ALL cases after RNA interference with SLC7A11 in MSC using 2 individual siRNA sequences identified a subset of ALL cases that depends on stromal SLC7A11.

To identify critical determinants of stromal support in our model, we customized an siRNA selection of 110 candidate genes for screening (Supplemental Table S2). These candidates were selected based on the following filtering criteria: (i) presence on MSC by cell surface capturing proteomics (Supplemental Table S2); (ii) detection by gene expression profiling data (Affymetrix human gene 1.0 ST); (iii) functional annotations indicative of a role in cell adhesion, cell-to-cell signalling, interactions with the extracellular matrix, or in a hematopoietic stem cell niche function; (iv) comparative analyses with cell surface proteome data of ALL cells [21], indicating whether or not putative interaction partners are detected in ALL cells. Since we expected to detect distinct patterns for different patients, we proceeded in two steps. We first performed a screen in triplicates on three cases with very high risk (VHR) ALL based on persistence of MRD [16] (VHR-01, VHR-03 and VHR-04) and detected distinct cell viability patterns in each case. Overall, 56 out of 330 siRNA transfections (3 patients × 110 RNAi) resulted in a significant decrease in ALL viability (Mann-Whitney U test), indicating that multiple determinants are involved (Fig. 3A, Supplemental Table S3). We did not detect any of these stromal genes alone to significantly confer universal support activity for these three ALL samples. However, RNA interference with 20 of these 110 candidate genes in MSC significantly affected ALL survival in two of the three patient samples (Fig. 3A, top 20 genes of 1^st^ screen, Supplemental Table S3). To identify the most common determinants we extended the analysis to a cohort of 10 patients (Fig. 3A, 2^nd^ screen). Interference with any of these 20 genes did not affect MSC viability or morphology based on viability and actin filament stainings. A significant effect on ALL cell viability could be detected for 9 out of 10 ALL samples screened (Fig. 3A, 2^nd^ screen). The contribution of the individual candidate stromal genes varied for each leukemia sample. These cues included soluble and membrane-bound mediators of stromal support. Some of the hits included genes that contribute to the normal hematopoietic niche function, such as components of the WNT/beta-catenin pathway and the NOTCH pathway. For others, direct or indirect evidence for interactions with ALL cells have been reported. VCAM1 mediated stroma-ALL interactions through the binding of VLA-4 [4, 22]. The cytokine TSLP stimulates a pathway that is frequently mutated in BCP-ALL [23–24], suggesting that stromal TSLP could be relevant in this context.

### The stromal cystine transporter (x_c_^−^) contributes to ALL support

The strongest loss in ALL viability across these 10 samples was observed after down-regulation of solute carrier family 3 member 2 (SLC3A2) in MSC (Fig. 3A, 2^nd^ screen). This effect was confirmed in independent experiments using flow cytometry to assess viability (Fig. 3B). Interestingly, a subset of the ALL cases tested was highly sensitive to interference with SLC3A2. As the imaging screen was performed with pools of four siRNAs for each target, we deconvoluted this pool and confirmed these results using four single siRNA sequences (Fig. 3C).

The cell membrane protein SLC3A2 is a member of the solute carrier family proteins and constitutes the large subunit of heterodimeric amino acid transporters (HATs). Together with SLC7A11, it forms the x_c_^−^ cystine/glutamate antiporter for cellular uptake of cystine in exchange for intracellular glutamate [25]. A role for stromal cystine metabolism was recently reported to support chronic lymphoblastic leukemia (CLL) [26]. To verify that cystine uptake by MSC accounts for stromal SLC3A2 dependency, we supplemented cystine-depleted medium with increasing concentrations of cystine in co-culture experiments. As expected, SLC3A2-dependent cases were highly sensitive to cystine depletion, and this effect could be rescued in a dose-dependent manner by cystine supplementation (Fig. 3D). Thus, cystine supply is a limiting factor especially essential for the survival of stromal SLC3A2 dependent cases.

To confirm if functional x_c_^−^ in MSC account for the support to ALL cells, we targeted the second subunit of x_c_^−^ (SLC7A11) in MSC with 2 individual siRNAs in a set of 27 primary ALL cases, which recapitulated the phenotype of RNA interference with SLC3A2 (Fig. 3E). Around one third of the samples tested lost more than fifty percent of the viability upon perturbation of stromal SLC7A11 function in this model. In contrast to the general pattern of dependence on stromal cystine metabolism reported for CLL [26], this phenotype appears to be restricted to a subset in ALL. We did not detect any obvious association with clinical or genetic characteristics (drug resistance phenotype, genetic abnormalities, Supplementary Fig. S1A). Furthermore, the relative levels of protein detected by western blot for SLC7A11 was not reduced in ALL compared to MSC (Supplementary Fig. S1B), which suggests that unlike described for CLL [26], intrinsic metabolic deregulation rather than x_c_^−^ dysfunction account for this dependency pattern. Taken together, our experiments show that the cystine transporter in MSC is essential for the survival of a subset of ALL cases, which we will refer to herein as stromal x_c_^−^-dependent.

### Stromal x_c_^−^ activity protects ALL from oxidative stress

**Figure 4:**
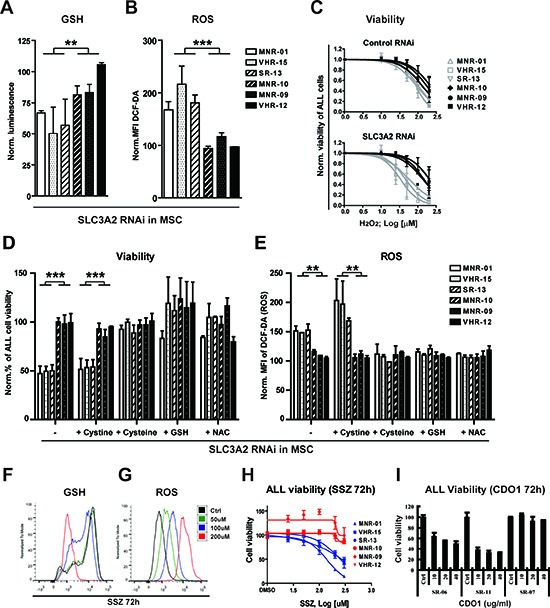
Stromal cysteine supply is required to control oxidative stress in ALL samples that are dependent on stromal SLC3A2/SLC7A11 Comparison of the GSH **(A)** and ROS levels **(B)** of patient samples that are dependent (n = 3) and independent (n = 3) on stromal SLC3A2 assessed after RNA interference with SLC3A2 in MSC after 3 days of co-culture. **(C)** Sensitivity to H_2_O_2_ was decreased in the 3 SLC3A2 dependent samples but not in the 3 independent samples after RNA interference with stromal SLC3A2. **(D)** The stromal support function lost after RNA interference with SLC3A2 is rescued and **(E)** ROS levels are restored to control levels by the addition of cysteine (100 μM), GSH (5 mM) or NAC (5 mM), but not cystine (100 μM). ALL cell viability and ROS levels (DCF-DA) were assessed by flow cytometry after 6 days of co-culture. **(F and G)** GSH and ROS levels were assessed by flow cytometry after incubation with SSZ for 72 hours in the stromal x_c_^−^-dependent ALL sample SR-13. **(H)** SSZ decreased ALL cell viability of 3 stromal x_c_^−^ dependent ALL samples (blue curve) while 3 independent samples (red curve) remained largely unaffected after 3 days incubation with SSZ in coculture. **(I)** Recombinant human cysteine dioxygenase (CDO1) decreased the viability of x_c_^−^ dependent ALL samples. In contrast, x_c_^−^ independent ALL was not affected by CDO1 treatment. Histograms in A, B, C, D, E, H and I show the mean ± SD of values normalized to the respective negative controls (*P < 0.05; **P < 0.01; ***P < 0.001, Mann-Whitney U-test).

Once inside a cell, cystine can be reduced to cysteine, which constitutes a major source for gluthathione (GSH) synthesis. Given the dependence of CLL cells on stromal cysteine supply to maintain GSH synthesis and mitigate oxidative stress [26], we sought to evaluate the contribution of stromal x_c_^−^ to the control of oxidative stress in ALL cells. RNA interference with SLC3A2 resulted in a decrease of GSH in MSC, which did not affect their viability (data not shown). Comparing three stromal x_c_^−^-dependent and three stromal x_c_^−^ -independent cases, RNA interference with stromal SLC3A2 resulted in a marked decrease of GSH and an increase of ROS in stromal x_c_^−^-dependent cases (Fig. 4A, B). Thus, SLC3A2-dependent ALL appears to be dependent on stromal x_c_^−^ function to maintain GSH levels and regulate ROS. Consistently, the stromal x_c_^−^-dependent ALL cells are more sensitive to exogenous oxidative stress by hydrogen peroxide, an effect that was further pronounced by interference with SLC3A2 in stromal cells (Fig. 4C). To confirm that reduction of cystine to cysteine in MSC was required for this mechanism of support, we could show that supplementation of the medium with cysteine, but not cystine could rescue the effect of RNA interference with SLC3A2 in MSC on ALL survival (Fig. 4D, E). The same results were obtained using another human stromal cell line, HS-5 (Supplementary Fig. S2). Moreover, supplementation of the medium with GSH or addition of the antioxidant N-acteylcysteine (NAC) rescued the effect of SCL3A2 RNA interference (Fig. 4D, E). Collectively our findings demonstrate that the conversion of cystine to cysteine by MSC sustains GSH levels and reduces oxidative stress, which are critical components of the stromal support mechanism.

### The pharmacological depletion of cysteine in microenvironment compromises survival of ALL cases that are dependent on stromal x_c_^−^

Selective metabolic dependence on this antioxidant pathway provides a new rationale for therapeutic intervention. As a proof of concept, we used Sulfasalazine (SSZ), an antiinflammatory agent that is commonly used in the clinic and which was show to block x_c_^−^ activity [27]. Treatment of ALL-MSC co-cultures with SSZ lead to a potent GSH depletion and ROS overload in the stromal x_c_^−^-dependent ALL samples (Fig. 4F, G). This was achieved at a concentration that did not affect the MSC compartment. As expected, stromal x_c_^−^-dependent ALL cases were more sensitive to SSZ than x_c_^−^-independent ALL cases (Fig. 4H). In line with a critical and selective role for stromal cysteine in ALL support, treatment of co-cultures with recombinant cysteine dioxygenase (CDO1), which catalyzes conversion of cysteine into cysteine sulfinic acid and bypasses GSH synthesis [28], reduced the survival of x_c_^−^-dependent ALL cells, while x_c_^−^-independent ALL cells did not respond to this treatment (Fig. 4I). Such metabolic vulnerability may be exploited to develop more specific agents in order to increase sensitivity to antileukemic therapy.

## DISCUSSION

Interactions between leukemia cells and their microenvironment have important effects on critical cellular programs in cancer cells, with potential implications for disease propagation and drug resistance [29]. Systematic investigation using cancer cell lines indicate that stroma mediated resistance to targeted agents could be common [30]. Moreover, altered stromal function can favour leukemogenic events and influence disease progression [31–32]. Our current knowledge about molecular determinants of the leukemia niche is still very limited. To facilitate large-scale functional investigation in an *in vitro* model of the niche, we tailored an image-based screening platform using primary leukemia patient samples on bone marrow mesenchymal stromal cells. This approach enables to screen primary ALL cells directly without having to generate gene-marked lines. Elegant compartment-specific screens have provided important insights into stromal mediated drug resistance mechanisms, but so far have been limited to estabslished cell lines [3, 30]. Our microscopy-based platform is suitable for large scale functional profiling using genetic or pharmacologic interference. We provide an open source modular image analysis pipeline with an efficient algorithm based on machine learning to discriminate the two cellular compartments in this model, which can easily be incorportated in other experimental settings. This approach captured significant individual differences between leukemia samples with respect to the stroma support function. With the development of new methods for genetic interference such as lentiviral CRISPR-Cas9 knockout technology [33], our platform will enable compartment specific synthetic lethality interrogation in relevant patient samples.

Most of the ALL cases that we evaluated depended on MSC for survival *in vitro*, even in presence of exogenous serum factors. This provides a model to study the bidirectional interactions that may occur in the leukemia niche. We recognize that this *in vitro* model has limitations, but it is currently impossible to interefere with stromal function in a systematic approach in vivo using a model that is based on human leukemia samples. We choose mesenchymal stromal cells for our model based on several considerations. Lineage mapping studies based on the activity of developmental specific promoter activity (nestin and prx1 promoters) in mice identified overlapping subsets of mesenchymal progenitors important for normal HSC and lymphoid progenitor maintenance [5, 10]. These bone marrow perivascular mesenchymal stem cells are an important component of the niche and contribute to its formation [7]. Mesenchymal stromal cells are a component of the ALL niche in the xenograft model [2]. Explanted MSC have the capacity to organize and reconstitute the hematopoietic niche *in vivo* [5, 7]. The hTERT-immortalized MSC that were used for our study display high PRRX1 RNA levels (data not shown) and fulfill progenitor-defining criteria [12].

**Figure 5:**
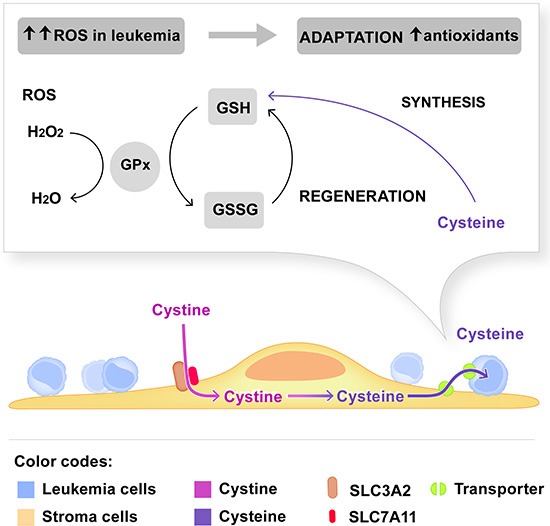
Model depicting the dependence of leukemia cells on cystine metabolism of stromal cells The stromal cystine transporter composed of SLC3A2 and SLC7A11 is required for the import of cystine in MSC. Reduction of cystine to cysteine and subsequent supply to leukemia cells are critical steps for GSH synthesis in leukemia cells. GSH is an important antioxidant to maintain the redox state in leukemia cells that generate more ROS than non-malignant cells. The experimental evidence is summarized in the discussion.

Metabolic reprogramming is a dominant feature of cancer cells [34]. Metabolite profiling of leukemia cells confirmed their increased biosynthetic activity [35]. This requires an adaptation of cancer cells to produce sufficient anti-oxidants such as NADPH [36] and reduced glutathione [34]. Here we identified a mechanism for specific metabolic support of glutathione synthesis in ALL that depends on the function of the cystine transporter (SLC3A2/SLC7A11) on MSC cells. SLC3A2 together with SLC7A11 forms a HAT that can exchange cystine for glutamate. Our data support a model in which SLC3A2/SLC7A11 is essential to mediate import of cystine into MSC as a first step (Fig. 5). Cystine import into stroma and reduction to cysteine is required for ALL support, because loss of stromal SLC3A2 is rescued by supplementation with cysteine but not with cystine. Cysteine is a rate limiting substrate for GSH synthesis together with glycine and glutamine. Because reduced cysteine is rapidly oxidized in the microenvironment, constant supply for ALL cells appears to be needed for the metabolic control of the redox state in ALL. Indeed, we show that interference with the cystine-cysteine supply is associated with a significant decrease of GSH and concomitant increase in ROS, which results in marked induction of apoptosis only in ALL samples that are dependent of this stromal function of SLC3A2. Our results extend the recent study in CLL [26] and indicate that exogenous cysteine supply may also be critical for acute leukemia. But we show here that this interdependence varies individually, with a subset of tested ALL cases that are highly dependent on cysteine supply. For ALL, this phenotype is not explained by a difference in HAT levels (Supplementary Fig. S1B), in contrast to the possible correlation that was reported for CLL [26]. Thus, major underlying metabolic deregulation in ALL rather than differential HAT expression account for the vulnerability to cysteine depletion. The underlying mechanisms remain to be explored. Given the possibilities to target the oxidative stress pathway directly [26] or through depletion of NADPH [37], it will be interesting to systematically screen for bioactive and therapeutic agents in this context.

Taken together, our data illustrate that distinct networks of interactions between leukemia cells and MSC exist, which has implications for the development of targeted therapies. Metabolic support may constitute a predominant function of the leukemia niche, as reflected by the selective dependence on stromal cysteine to control ROS in leukemia cells. This metabolic vulnerability could be exploitable therapeutically. Our experiments indicate that targeting cysteine dioxygenase may increase ALL vulnerability in a selected way. Similarly, the dependence of ALL on asparagine provides the basis for therapy with asparaginase, which can also be influenced by the metabolic activity of the niche [38]. As lesions in metabolic networks in leukemia are being increasingly identified, it will be essential to understand their implications in the context of the tumor microenvironment. Functional profiling approaches such as the one shown here will contribute to detect disease specific druggable targets in a more individualized manner.

## METHODS

### Patient samples

Cryopreserved bone marrow samples from patients who had given informed consent in accordance with the Declaration of Helsinki were used for xenotransplantation in NOD-scid-IL2Rg(−/−) (NSG) mice as described [15–16]. Cells from second and third passage in NSG mice were recovered from spleens (>95% of human ALL cells) and used for in vitro experiments (Supplemental Table 1). Samples from first presentation were from patients on the trial ALL-BFM 2000 [17] stratified in standard risk (SR), medium risk (MR), high risk (HR) and very high risk (VHR) of relapse by MRD. Samples from morphological non responder patients (MNR) were obtained at relapse on the ALL-REZ-BFM 2002 study [39]. MNR was defined as absence of morphological remission after the second block of chemotherapy on this protocol.

### Cell culture

Human *hTERT*-immortalized primary bone marrow MSC were provided by D. Campana (St Jude Children's Research Hospital, Memphis, Tennessee) and maintained in RPMI-1640, L-glutamine, 10% fetal bovine serum (Sigma) and 1 μM Hydrocortisone. HS-5 cells were purchased from ATCC (CRL-11882). Cystine, cysteine, GSH, N-acetylcysteine and SSZ were all from Sigma. Recombinant human cysteine dioxygenase (CDO1) was from Novus. MSC and ALL cells in co-culture were cultivated in AIM-V medium (Life technologies) with a ratio of 1:10 at indicated compound concentrations. In 384 well plates, 25 000 ALL cells were seeded on 2 500 MSC per well. For HS-5 co-cultures a ratio of 1:20 was used. Transwells® of 0.4 μ pore size (Corning) were used for the transwell assays. Cystine depleted RPMI-1640 medium was purchased from Sigma.

### RNAi screen and validation assays

The custom library (ON-TARGETplus siRNAs with 4 siRNAs per gene) was purchased from Thermo Fisher Scientific. For the screen, the 4 siRNA sequences were pooled to a final siRNA concentration of 30 nM. For validation experiments the 4 single siRNAs for each target were tested individually at a concentration of 20 nM. Reverse transfection was performed using the INTERFERin® reagent (Polyplus-Transfection SA) according to the manufacturer's instructions. For all validation assays, RT-PCR was performed to check for RNA interference at mRNA level of the targeted genes.

### Microscopy and multi-parametric image analysis

Co-cultures of MSC and ALL cells were stained with CyQUANT® Direct Cell Proliferation Assay (Life Technologies). The mixture of component A (diluted to 1:1200 in AIM-V medium) and component B (diluted to 1:80 in AIM-V medium) was added to the cells for 60 minutes at 37°C and 5% CO_2_ prior imaging. Images were taken on the ImageXpress Micro microscope (Molecular Devices) with a CoolSNap HQ camera (Photometrics) and a 0.3 NA 10x Plan Fluor objective (Nikon). Image acquisition was set to cover more than 50% of each well in a total of 9 images (Fig. 2B).

For analysis, images were first segmented using the CellProfiler software [19] and distinction of viable ALL cells from dead ALL cells as well as MSC was implemented in a machine learning tool for high-content analysis (Advanced Cell Classifier, ACC) [20]. An ‘a trous’ wavelet transform-based spot detection algorithm [18] was built into the CellProfiler framework. For each detected cell, over 50 intensity and texture-based features were extracted. The list of extracted features, CellProfiler pipelines, and the parallelization code are accessible at www.highcontentanalysis.org. For correct assignment of ALL cells, the open source Advanced Cell Classifier program [20] was used with the random forest classification method for all experiments [40].

### Flow cytometry

Cell viability assays were performed as described [12, 15] using 7-AAD (BD Pharmingen) and anti-human CD45 (FITC) or CD19 (PE) (AbD Serotec) antibodies.

### Western blot

Whole-cell extracts were prepared from 1 × 10^6^ cells using (RIPA) buffer (50 mM Tris-Cl, pH 6.8, 100 mM NaCl, 1%Triton-X-100, 0.1% SDS) supplemented with complete mini protease inhibitor cocktail (Roche Applied Sciences) for 20 minutes on ice and processed according to standard protocols. Anti-SLC7A11 antibody (Novus NB300-318) was used for detection with chemiluminescence.

### Glutathione and ROS measurements

Glutathione levels were measured using the GSH-Glo™ Glutathione Assay (Promega). For measurements of ROS levels, cells were stained after 3 days in co-culture with 20 μM DCF-DA (Sigma) for 2 hours prior flow cytometry analysis.

### Statistic analysis

To compare cell viability, GSH level and ROS level in the SLC3A2 dependent patients and the SLC3A2 independent patients, the Mann-Whitney U-test was used. The p-values lower than 0.05 were considered statistically significant. Heatmaps were generated with R, and histograms and plots were generated using the Prism software (GraphPad).

## SUPPLEMENTARY FIGURES AND TABLES


