# Supplementary material for: Image-based RNA interference screening reveals an individual dependence of acute lymphoblastic leukemia on stromal cysteine support
Source: Oncotarget. 2014 Nov 8;5(22):11501–12. doi: 10.18632/oncotarget.2572 (PMC4294362; doi:10.18632/oncotarget.2572)
Supplement: Supplementary file 1 [file oncotarget-05-11501-s001.pdf]

SUPPLEMENTARY FIGURES AND TABLES

A

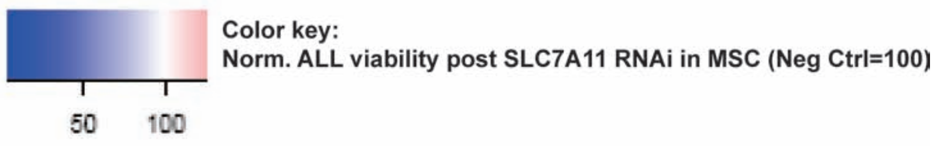

ID	Translocation / Karyotype	Prednisolone response	Relapse
SR.06	high hyperdiploid	good	relapse
SR.11	na	good	relapse
VHR.15	9p del.	good	na
SR24	t(1;19)	poor	na
SR.10	na	good	na
SR.13	na	good	na
VHR.10	na	poor	na
VHR.07	t(4;11)	poor	na
MNR.01	TP53 del.	na	relapse
VHR.04	na	good	na
SR.02	na	good	na
HR.20	t(17;19)	good	relapse
SR.22	t(1;19)	poor	na
HR.24	t(17;19)	na	relapse
MNR.10	na	na	relapse
HR.21	t(17;19)	good	relapse
VHR.02	na	poor	relapse
VHR.03	na	poor	na
VHR.01	t(17;19)	poor	relapse
HR.03	t(9;22), high hyperdiploid	good	na
VHR.06	t(1;19)	good	relapse
SR.04	t(1;19)	good	na
VHR.12	na	na	na
MNR.09	TP53,WT	na	relapse
SR.07	na	good	na
MR.01	t(1;19)	poor	na
SR.23	t(1;19)	good	na

B

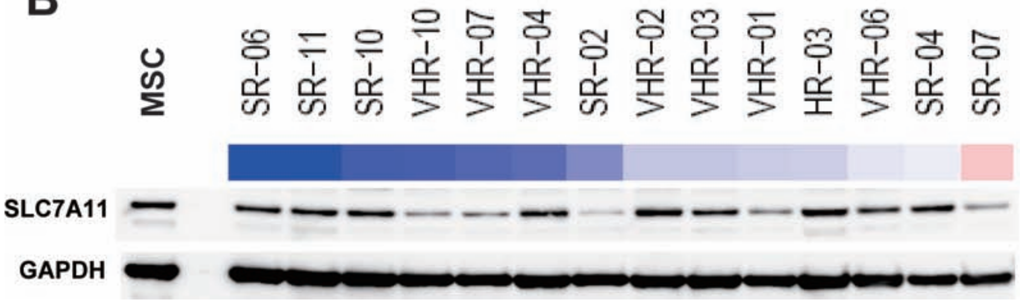

Supplementary Figure S1: (A) ALL samples were tested for their dependency on stromal SLC7A11. The known genetic abnormalities, drug resistance phenotypes and occurrence of relapse are indicated. **(B)** Protein expression level of SLC7A11 in MSC and ALL samples with different dependency on stromal SLC7A11. The color code from blue to red in (A) and (B) indicates decreasing dependency on stromal SLC7A11.

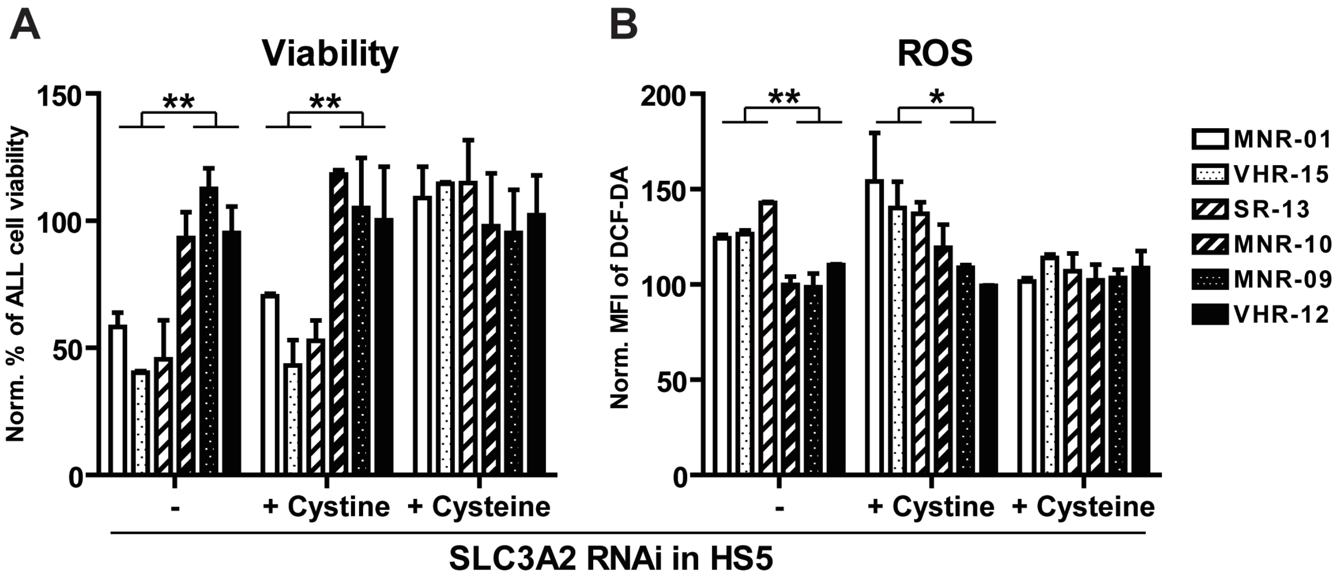

Supplementary Figure S2: ALL cell viability (A) and ROS level **(B)** assessed by flow cytometry for 3 SLC3A2-dependent and 3 SLC3A2-independent cases after RNA interference with SLC3A2 in the human stromal cell line HS-5 cultured in normal (-), 100 µM cystine supplied (+ Cystine) or 100 µM cysteine supplied (+ Cysteine) conditions after 6 (A) or 3 (B) days of co-culture. Histograms show the mean ± SD of values normalized to the respective negative controls (*$P < 0.05$; **$P < 0.01$, Mann-Whitney U-test).

Supplementary Table S1. ALL patient characteristics.

Patient	Immunology	Age at initial diagnosis	Sex	Prednisone response	Risk group	Relapse	Survival	Leukocyte count	Blasts periph %	Additional information
SR-02	Pre-B-ALL	12.1	m	PGR	SR	no	yes	447000	94	Amplification AML1
SR-04	Common-ALL	3.6	f	PGR	SR	no	yes	10600	42	t(1;19)
SR-09	Common-ALL	5.5	f	PGR	SR	yes	yes	317400	na	
SR-11	Common-ALL	6.1	m	PGR	SR	yes	yes	66600	87	
SR-13	Common-ALL	4.1	f	PGR	SR	no	yes	21000	na	
HR-03	Common-ALL	16.9	f	PGR	HR	no	yes	13300	78	CNS, t(9;22), hhd
VHR-01	Common-ALL	14.1	m	PPR	VHR	yes, 2x	no	6300	13	early relapse, t(17;19)
VHR-02	Common-ALL	12.8	f	PPR	VHR	yes	yes	79600	68	
VHR-03	Common-ALL	17.1	m	PPR	VHR	no	no	20100	78	TRM
VHR-04	Common-ALL	5.7	f	PGR	VHR	no	yes	45700	97	
VHR-06	Pre-B-ALL	17.6	m	PGR	VHR	yes	no	8000	73	complex karyotype, t(1;19)
VHR-10	Common-ALL	14.4	f	PPR	VHR	no	yes	150000	89	
VHR-11	Pre-B-ALL	3.1	m	PGR	VHR	yes	no	16000	na	DS
VHR-12	Pre-B-ALL	15.3	f	PPR	VHR	no	yes	7412	92	
VHR-15	Pre-B-ALL	13.2	m	PGR	VHR	yes	yes	2900	60	deletion 9p
VHR-28	Common-B-ALL	na	f	na	VHR	yes	na	na	na	
MNR-01	Pre-B-ALL	9.6	f	PGR	MNR	yes	no	2346	na	TP53 del.
MNR-03	Pre-B-ALL	4.3	f	PPR	MNR	yes	no	64410	na	
MNR-04	Pre-B-ALL	9.8	m	PPR	MNR	yes	no	5040	na	
MNR-07	Pre-B-ALL	12.6	f	PPR	MNR	yes	no	27945	na	t(4;11)
MNR-09	Pre-B-ALL	3.3	m	PPR	MNR	yes	no	250	na	
MNR-10	Pre-B-ALL	9.7	m	PPR	MNR	yes	no	4165	na	

Risk group assessed accordingly on the MRD result:
patients are defined here as standard risk (SR) if MRD1 + 2 were negative, high risk (HR) if MRD1 + 2 were positive less than or equal to 0.001, and VHR if HR patients were still positive for MRD3
MNR stand for morphological non-responder, patients with relapse ALL
CNS: disease spread into central nervous system
hhd: hyperdiploid
TRM: treatment-related mortality
na: not available

Supplementary Table S2A. Proteins on MSCs identified by mass spectrometry.

Entrez gene symbol	Entrez Gene ID	CD annotation	Number of distinct peptides	Assignment to membrane	UniProtKB/ Swiss-Prot ID	UniProtKB/ Swiss-Prot AC2
ABCA8	10351	na	1	1	ABCA8_ HUMAN	O94911
ACE	1636	CD143	1	1	ACE_ HUMAN	P12821
ACTA2	59	na	1	0	ABCC8_ HUMAN	Q09428
ACTB	60	na	2	0	ACTB_ HUMAN	P60709
ADAM15	8751	na	1	1	PCTK3_ HUMAN	Q07002
ADAM17	6868	CD156b	1	1	ADA17_ HUMAN	P78536
ADAM9	8754	na	1	1	PCY1A_ HUMAN	P49585
ADCY9	115	na	2	1	ADCY9_ HUMAN	O60503
AGGF1	55109	na	1	0	AGGF1_ HUMAN	Q8N302
AHSG	197	na	1	0	FETUA_ HUMAN	P02765
ALB	213	na	2	0	ALBU_ HUMAN	P02768
ALCAM	214	CD166	22	1	ADAM7_ HUMAN	Q9H2U9
ALDOA	226	na	1	1	RED1_ HUMAN	P78563
ALPL	249	na	11	0	PPBT_ HUMAN	P05186
ALS2CL	259173	na	1	0	AL2CL_ HUMAN	Q60I27
ANKFY1	51479	na	1	0	ANFY1_ HUMAN	Q9P2R3
ANPEP	290	CD13	8	1	AMPN_ HUMAN	P15144
ANTXR1	84168	na	1	1	ANTR1_ HUMAN	Q9H6X2
ASAM	79827	na	2	1	ASAM_ HUMAN	Q9H6B4
ATP1B3	483	CD298	3	1	AT1B3_ HUMAN	P54709

(*Continued*)

Entrez gene symbol	Entrez Gene ID	CD annotation	Number of distinct peptides	Assignment to membrane	UniProtKB/ Swiss-Prot ID	UniProtKB/ Swiss-Prot AC2
AUTS2	26053	na	1	0	AUTS2_ HUMAN	Q8WXX7
AXUD1	64651	na	1	0	AXUD1_ HUMAN	Q96S65
BAT2D1	23215	na	1	0	PIGO_ HUMAN	Q8TEQ8
BRDG1	26228	na	1	0	STAP1_ HUMAN	Q9ULZ2
BSG	682	CD147	4	1	BASI_ HUMAN	P35613
BTN3A3	10384	na	1	1	RS11_ HUMAN	P62280
C14orf78	113146	na	2	1	AHNK2_ HUMAN	Q8IVF2
C19orf31	404664	na	1	0	na	na
C5	727	na	1	0	ARTN_ HUMAN	Q5T4W7
CACNA1C	775	na	1	1	CAC1C_ HUMAN	Q13936
CACNA2D1	781	na	3	1	CAC2D_ HUMAN	P54289
CASQ2	845	na	1	0	CASQ2_ HUMAN	O14958
CD109	135228	CD109	4	1	CD109_ HUMAN	Q6YHK3
CD151	977	CD151	4	1	CD151_ HUMAN	P48509
CD248	57124	CD248	2	1	CD248_ HUMAN	Q9HCU0
CD276	80381	CD276	2	1	CD276_ HUMAN	Q5ZPR3
CD44	960	CD44	6	1	CD44_ HUMAN	P16070
CD55	1604	CD55	3	1	DAF_ HUMAN	P08174
CD59	966	CD59	23	1	CD59_ HUMAN	P13987
CD63	967	CD63	2	1	CD63_ HUMAN	P08962
CDC42	998	na	2	0	BCL3_ HUMAN	P20749

(*Continued*)

Entrez gene symbol	Entrez Gene ID	CD annotation	Number of distinct peptides	Assignment to membrane	UniProtKB/ Swiss-Prot ID	UniProtKB/ Swiss-Prot AC2
CDH2	1000	CD325	2	1	CADH2_HUMAN	P19022
CDSN	1041	na	1	0	BFSP2_HUMAN	Q13515
CFP	5199	na	1	0	PROP_HUMAN	P27918
CHD9	80205	na	1	1	CHD9_HUMAN	Q3L8U1
CLIC4	25932	na	1	0	CLIC4_HUMAN	Q9Y696
CORIN	10699	na	1	1	CORIN_HUMAN	Q9Y5Q5
CPM	1368	na	3	0	CAH1_HUMAN	P00915
CRELD1	78987	na	1	1	CREL1_HUMAN	Q96HD1
CSHL1	1444	na	1	1	CSHL_HUMAN	Q14406
CSRP1	1465	na	1	0	CSRP1_HUMAN	P21291
CTGLF1	119016	na	1	0	CTLF1_HUMAN	Q96P64
CTSB	1508	na	1	0	CATB_HUMAN	P07858
CYB5R3	1727	na	1	0	NB5R3_HUMAN	P00387
DAG1	1605	na	1	1	CCR4_HUMAN	P51679
DCD	117159	na	4	0	DCD_HUMAN	P81605
DENND1B	163486	na	1	1	DEN1B_HUMAN	Q6P3S1
DNA2L	1763	na	1	0	DNA2L_HUMAN	P51530
DTNBP1	84062	na	1	0	DTBP1_HUMAN	Q96EV8
DVL1	1855	na	1	0	DVL1_HUMAN	O14640
DYX1C1	161582	na	1	1	DYXC1_HUMAN	Q8WXU2

(*Continued*)

Entrez gene symbol	Entrez Gene ID	CD annotation	Number of distinct peptides	Assignment to membrane	UniProtKB/ Swiss-Prot ID	UniProtKB/ Swiss-Prot AC2
EGFR	1956	na	5	1	EGFR_ HUMAN	P00533
EMP3	2014	na	2	1	EMP3_ HUMAN	P54852
EMR2	30817	CD312	1	1	EMR2_ HUMAN	Q9UHX3
ERBB2	2064	CD340	1	1	ERBB2_ HUMAN	P04626
ERLIN1	10613	na	1	0	ERLN1_ HUMAN	O75477
FARP1	10160	na	1	0	FARP1_ HUMAN	Q9Y4F1
FAS	355	CD95	2	1	TNR6_ HUMAN	P25445
FAT	2195	na	1	1	COIA1_ HUMAN	P39060
FBXL20	84961	na	1	0	FXL20_ HUMAN	Q96IG2
FHL2	2274	na	1	0	FHL2_ HUMAN	Q14192
FLJ21963	79611	na	1	0	ACSS3_ HUMAN	Q9H6R
FLJ34931	388939	na	1	0	CB071_ HUMAN	A6NGG8
FLNA	2316	na	4	0	FLNA_ HUMAN	P21333
FN1	2335	na	23	1	FINC_ HUMAN	P02751
GAK	2580	na	1	0	GAK_ HUMAN	O14976
GAS2L3	283431	na	1	0	GA2L3_ HUMAN	Q86XJ1
GCC2	9648	na	1	0	GCC2_ HUMAN	Q8IWJ2
GGA2	23062	na	1	0	GGA2_ HUMAN	Q9UJY4
GGTLA1	2687	na	4	0	GGT5_ HUMAN	P36269
GJA1	2697	na	1	1	DBP_ HUMAN	Q10586

(*Continued*)

Entrez gene symbol	Entrez Gene ID	CD annotation	Number of distinct peptides	Assignment to membrane	UniProtKB/ Swiss-Prot ID	UniProtKB/ Swiss-Prot AC2
GNAI2	2771	na	2	1	GNAI2_ HUMAN	P04899
GNB1	2782	na	1	0	GBB1_ HUMAN	P62873
GPRIN3	285513	na	1	0	GRIN3_ HUMAN	Q6ZVF9
GTF2IRD1	9569	na	1	0	GT2D1_ HUMAN	Q9UHL9
		na	3	0	K2C8_ HUMAN	P05787
HLA-A	3105	na	3	1	multiple	multiple
HLA-B	3106	na	4	1	multiple	multiple
HLA-C	3107	na	2	1	multiple	multiple
HSPG2	3339	na	8	0	PGBM_ HUMAN	P98160
ICAM1	3383	CD54	1	1	ICAM1_ HUMAN	P05362
IGF2R	3482	CD222	1	1	MPRI_ HUMAN	P11717
INSRR	3645	na	1	1	INSRR_ HUMAN	P14616
ISCU	23479	na	1	1	ISCU_ HUMAN	Q9H1K1
ITGA11	22801	na	1	1	ITA11_ HUMAN	Q9UKX5
ITGA3	3675	CD49c	6	1	ITA3_ HUMAN	P26006
ITGA5	3678	CD49e	23	1	ITA5_ HUMAN	P08648
ITGAV	3685	CD51	11	1	ITAV_ HUMAN	P06756
ITGB1	3688	CD29	16	1	ITB1_ HUMAN	P05556
ITGB5	3693	na	2	1	ITB5_ HUMAN	P18084
KALRN	8997	na	1	0	KALRN_ HUMAN	O60229
KIAA1546	54790	na	1	0	TET2_ HUMAN	Q6N021

(Continued)

Entrez gene symbol	Entrez Gene ID	CD annotation	Number of distinct peptides	Assignment to membrane	UniProtKB/ Swiss-Prot ID	UniProtKB/ Swiss-Prot AC2
KIF12	113220	na	1	0	KIF12_ HUMAN	Q96FN5
KIF1B	23095	na	1	0	KIF1B_ HUMAN	O60333
KRT1	3848	na	9	0	K2C1_ HUMAN	P04264
KRT10	3858	na	13	0	K1C10_ HUMAN	P13645
KRT2	3849	na	16	0	K22E_ HUMAN	P35908
KRT4	3851	na	3	1	K2C4_ HUMAN	P19013
KRT5	3852	na	2	0	K2C5_ HUMAN	P13647
KRT6A	3853	na	1	0	K2C6A_ HUMAN	P02538
KRT6B	3854	na	4	0	K2C6B_ HUMAN	P04259
KRT8	3856	na	4	0	K2C8_ HUMAN	P05787
KRT9	3857	na	12	0	K1C9_ HUMAN	P35527
LAMC3	10319	na	1	0	LAMC3_ HUMAN	Q9Y6N6
LAMP1	3916	CD107a	1	1	LAMP1_ HUMAN	P11279
LEF1	51176	na	1	0	LEF1_ HUMAN	Q9UJU2
LEPR	3953	CD295	3	1	NCS1_ HUMAN	P62166
LMNA	4000	na	1	0	LMNA_ HUMAN	P02545
LOC644196	644196	na	1	0	na	na
LOC645745	4496	na	1	0	na	na
LRP1	4035	CD91	18	1	LRP1_ HUMAN	Q07954
LRRC16	55604	na	1	0	LR16A_ HUMAN	Q5VZK9
LYST	1130	na	1	0	LYST_ HUMAN	Q99698

(Continued)

Entrez gene symbol	Entrez Gene ID	CD annotation	Number of distinct peptides	Assignment to membrane	UniProtKB/ Swiss-Prot ID	UniProtKB/ Swiss-Prot AC2
M6PR	4074	na	1	1	MPRD_ HUMAN	P20645
MASP2	10747	na	1	0	MASP2_ HUMAN	O00187
MATR3	9782	na	1	0	MATR3_ HUMAN	P43243
MRC2	9902	CD280	1	1	MRC2_ HUMAN	Q9UBG0
MSN	4478	na	1	0	MOES_ HUMAN	P26038
MT1E	4493	na	1	0	MT1E_ HUMAN	P04732
MT1G	4495	na	2	1	MT1G_ HUMAN	P13640
MTE	644314	na	1	0	na	na
MTRR	4552	na	1	1	ALAT1_ HUMAN	P24298
MTSS1	9788	na	1	0	MTSS1_ HUMAN	O43312
MYBBP1A	10514	na	1	0	MBB1A_ HUMAN	Q9BQG0
MYEF2	50804	na	1	0	MYEF2_ HUMAN	Q9P2K5
NCDN	23154	na	1	0	NCDN_ HUMAN	Q9UBB6
NEGR1	257194	na	5	0	NEGR1_ HUMAN	Q7Z3B1
NEU4	129807	na	1	0	NEUR4_ HUMAN	Q8WWR8
NID2	22795	na	1	0	NID2_ HUMAN	Q14112
NOPE	57722	na	1	1	IGDC4_ HUMAN	Q8TDY8
NOTCH2	4853	na	2	1	NOTC2_ HUMAN	Q04721
NOTCH3	4854	na	1	0	NOTC3_ HUMAN	Q9UM47
NPR2	4882	na	1	1	ANPRB_ HUMAN	P20594
NPR3	4883	na	3	1	ANPRC_ HUMAN	P17342

(*Continued*)

Entrez gene symbol	Entrez Gene ID	CD annotation	Number of distinct peptides	Assignment to membrane	UniProtKB/ Swiss-Prot ID	UniProtKB/ Swiss-Prot AC2
NPTN	27020	na	2	1	NPTN_ HUMAN	Q9Y639
NRP1	8829	CD304	2	1	NRP1_ HUMAN	O14786
NT5E	4907	CD73	19	1	5NTD_ HUMAN	P21589
OTOF	9381	na	2	1	OTOF_ HUMAN	Q9HC10
P2RX4	5025	na	1	1	P2RX4_ HUMAN	Q99571
PAX2	5076	na	1	0	PAX2_ HUMAN	Q02962
PCCB	5096	na	1	0	PCCB_ HUMAN	P05166
PCDHGA1	56114	na	1	1	PCDG1_ HUMAN)	Q9Y5H4
PCF11	51585	na	1	0	PCF11_ HUMAN	O94913
PDGFRB	5159	CD140b	1	1	PGFRB_ HUMAN	P09619
PHLPP	23239	na	1	1	ZN473_ HUMAN	Q8WTR7
PKD2L1	9033	na	1	1	PK2L1_ HUMAN	Q9P0L9
PKHD1L1	93035	na	1	0	PKHL1_ HUMAN	Q86WI1
PLEKHH2	130271	na	2	0	PKHH2_ HUMAN	Q8IVE3
PLXNB2	23654	na	2	1	PLXB2_ HUMAN	O15031
PLXND1	23129	na	1	1	PLXD1_ HUMAN	Q9Y4D7
PPAP2B	8613	na	2	1	LPP3_ HUMAN	O14495
PRNP	5621	CD230	3	1	PRIO_ HUMAN	P04156
PSD3	23362	na	1	0	PSD3_ HUMAN	Q9NYI0
PTK7	5754	na	3	1	PTK7_ HUMAN	Q13308

(Continued)

Entrez gene symbol	Entrez Gene ID	CD annotation	Number of distinct peptides	Assignment to membrane	UniProtKB/ Swiss-Prot ID	UniProtKB/ Swiss-Prot AC2
PTPRM	5797	na	1	1	PTPRM_ HUMAN	P28827
PVRL2	5819	CD112	1	1	PVRL2_ HUMAN	Q92692
PVRL3	25945	CD113	2	1	PVRL3_ HUMAN	Q9NQS3
QRICH2	84074	na	2	0	QRIC2_ HUMAN	Q9H0J4
RAC1	5879	na	2	1	RAC1_ HUMAN	P63000
RAD17	5884	na	1	0	RAD17_ HUMAN	O75943
RECK	8434	na	18	1	RECK_ HUMAN	O95980
RFC1	5981	na	1	0	RFC1_ HUMAN	P35251
RHOQ	23433	na	1	0	RHOQ_ HUMAN	P17081
RNH1	6050	na	1	0	RINI_ HUMAN	P13489
RPA3	6119	na	1	0	RFA3_ HUMAN	P35244
RPL34	6164	na	1	0	RL34_ HUMAN	P49207
RPS12	6206	na	1	0	JUND_ HUMAN	P17535
RPS3	6188	na	1	0	RS3_ HUMAN	P23396
SALL1	6299	na	1	0	SALL1_ HUMAN	Q9NSC2
SCARF2	91179	na	1	0	SREC2_ HUMAN	Q96GP6
SCNN1B	6338	na	1	1	SCNNB_ HUMAN	P51168
SELP	6403	CD62	1	1	LYAM3_ HUMAN	P16109
SERPINB10	5273	na	1	0	SPB10_ HUMAN	P48595
SFRS2IP	9169	na	1	0	SFRIP_ HUMAN	Q99590

(*Continued*)

Entrez gene symbol	Entrez Gene ID	CD annotation	Number of distinct peptides	Assignment to membrane	UniProtKB/ Swiss-Prot ID	UniProtKB/ Swiss-Prot AC2
SIAH1	6477	na	1	1	SIAH1_HUMAN	Q8IUQ4
SLC1A4	6509	na	1	1	SATT_HUMAN	P43007
SLC2A1	6513	na	1	1	GTR1_HUMAN	P11166
SLC33A1	9197	na	1	1	ACATN_HUMAN	O00400
SLC39A14	23516	na	1	1	S39AE_HUMAN	Q15043
SLC3A2	6520	CD98	1	1	4F2_HUMAN	P08195
SLC44A1	23446	CD92	3	1	CTL1_HUMAN	Q8WWI5
SLC44A2	57153	na	3	1	CTL2_HUMAN	Q8IWA5
SMARCD2	6603	na	1	0	SMRD2_HUMAN	Q92925
SNED1	25992	na	1	0	SNED1_HUMAN	Q8TER0
SORT1	6272	na	1	1	KCNK1_HUMAN	O00180
SPTBN5	51332	na	1	0	SPTN5_HUMAN	Q9NRC6
STOML3	161003	na	1	1	STML3_HUMAN	Q8TAV4
tcag7.350	402694	na	1	0	na	na
TCEB3	6924	na	1	0	ELOA1_HUMAN	Q14241
TGFBR2	7048	na	1	1	TGFR2_HUMAN	P37173
THBS1	7057	na	1	0	CTGE5_HUMAN	O15320
THY1	7070	CD90	1	1	THY1_HUMAN	P04216
TNFRSF1B	7133	CD120b	1	1	TNR1B_HUMAN	P20333
TOM1L2	146691	na	1	0	TM1L2_HUMAN	Q6ZVM7
TPBG	7162	na	5	1	MMP16_HUMAN	P51512

(*Continued*)

Entrez gene symbol	Entrez Gene ID	CD annotation	Number of distinct peptides	Assignment to membrane	UniProtKB/ Swiss-Prot ID	UniProtKB/ Swiss-Prot AC2
TPM1	7168	na	2	0	TPM1_ HUMAN	P09493
TRIM25	7706	na	1	0	TRI25_ HUMAN	Q14258
TSPAN14	81619	na	1	1	TSN14_ HUMAN	Q8NG11
TSPAN4	7106	na	1	1	TSN4_ HUMAN	O14817
TTC7A	57217	na	1	0	TTC7A_ HUMAN	Q9ULT0
TTN	7273	na	1	0	TITIN_ HUMAN	Q8WZ42
TUBB2C	10383	na	1	0	TBB2C_ HUMAN	P68371
TXNDC4	23071	na	1	0	TXND4_ HUMAN	Q9BS26
UGT2B15	7366	na	1	1	UDB15_ HUMAN	P54855
VASN	114990	na	2	1	VASN_ HUMAN	Q6EMK4
VCAM1	7412	CD106	4	1	VCAM1_ HUMAN	P19320
VIM	7431	na	2	0	VIME_ HUMAN	P08670
VTI1B	10490	na	1	1	VTI1B_ HUMAN	Q9UEU0
ZBED5	58486	na	1	0	ZBED5_ HUMAN	Q49AG3
ZHX3	23051	na	1	0	ZHX3_ HUMAN	Q9H4I2
ZNF417	147687	na	1	0	ZN417_ HUMAN	Q8TAU3
ZPBP2	124626	na	1	0	ZPBP2_ HUMAN	Q6X784
ZYX	7791	na	1	1	ZYX_ HUMAN	Q15942

na - not assigned; assignment to the membrane based on predicted transmembrane domain and/or GPI-link and/or CD annotation; 0 - not assigned, 1- assigned

Supplementary Table S2B. 110 selected genes for siRNA screen.

Entrez gene symbol	CD annotation	Screen selected criteria	Biological property	Interacting proteins on ALL
CDC42BPA		GEP analysis	intracellular proteins	
CDC42BPB		GEP analysis	intracellular proteins	
CDGAP		GEP analysis	intracellular proteins	
MSN		GEP analysis	intracellular proteins	
RAC2		GEP analysis	intracellular proteins	
RAC3		GEP analysis	intracellular proteins	
RHOA		GEP analysis	intracellular proteins	
RHOB		GEP analysis	intracellular proteins	
RHOC		GEP analysis	intracellular proteins	
RHOG		GEP analysis	intracellular proteins	
RHOQ		GEP analysis	intracellular proteins	
CTNNB1		GEP analysis	beta-catenin signaling	
DKK1		GEP analysis	beta-catenin signaling	
DKK2		GEP analysis	beta-catenin signaling	
FZD6		GEP analysis	beta-catenin signaling	
FZD7		GEP analysis	beta-catenin signaling	
WISP1		GEP analysis	beta-catenin signaling	
ADAM15		MS-based screen	cell surface protein	ITGAV
ADAM17	CD156b	MS-based screen	cell surface protein	PDIA3,ADAM 10
ADAM9		MS-based screen	cell surface protein	ITGAV, ITGA6
ALCAM	CD166	MS-based screen	cell surface protein	CD6, ALCAM
ANPEP	CD13	MS-based screen	cell surface protein	RECK, MME, CD22
ANTXR1		MS-based screen	cell surface protein	LRP6, CD248, ANTRX2, PLXDC2
ASAM		MS-based screen	cell surface protein	
CD109	CD109	MS-based screen	cell surface protein	TGFB1
CD151	CD151	MS-based screen	cell surface protein	ITGB1, ITGA3,4,5 and 6
CD164L1	CD248	MS-based screen	cell surface protein	
CD276	CD276	MS-based screen	cell surface protein	CD58, ICAM1
CD44	CD44	MS-based screen	cell surface protein	ITGA4, SELL
CD55	CD55	MS-based screen	cell surface protein	EMR2, CD 47, 48, 58, 59, 97
CD59	CD59	MS-based screen	cell surface protein	CD9, EGFR, CD55
CD63	CD63	MS-based screen	cell surface protein	CD9, ITGB1, ITGAM
CDH2	CD325	MS-based screen	cell surface protein	ITGB1, PTPRC,J

(*Continued*)

Entrez gene symbol	CD annotation	Screen selected criteria	Biological property	Interacting proteins on ALL
EGFR		MS-based screen	cell surface protein	EGF
EMP3		MS-based screen	cell surface protein	P2RX7
ERBB2	CD340	MS-based screen	cell surface protein	PTPRC (CD45), PTPRJ
FAS	CD95	MS-based screen	cell surface protein	FASLG, CD47
FN1		MS-based screen	cell surface protein	ITGA5, CD79A, COL1A1
ICAM1	CD54	MS-based screen	cell surface protein	LFA-1, FGG, FGB, RPS27A,PTPRC
IGF2R	CD222	MS-based screen	cell surface protein	INSR, PLAUR
ITGA11		MS-based screen	cell surface protein	Integrins and MYH9
ITGA3	CD49c	MS-based screen	cell surface protein	CD9, ITGB1
ITGA5	CD49e	MS-based screen	cell surface protein	FN1, ITGB1,2,3
ITGAV	CD51	MS-based screen	cell surface protein	CD47, FN1, ITGB2,3
ITGB1	CD29	MS-based screen	cell surface protein	CD63, ITGB1, CD9
ITGB5		MS-based screen	cell surface protein	ITGA5
LAMP1	CD107a	MS-based screen	cell surface protein	TFRC, M6PR, CLTCL1, NCSTN,CD63
LEPR	CD295	MS-based screen	cell surface protein	PTPRC, PTPRJ
LRP1	CD91	MS-based screen	cell surface protein	PDGFRB, CALR, HSP90B1, THBS1
NRP1	CD304	MS-based screen	cell surface protein	PLXNA1, NGFR
NT5E	CD73	MS-based screen	cell surface protein	ENTPD1, ADA, FN1
P2RX4		MS-based screen	cell surface protein	H3F3A, P2RX7
PDGFRB	CD140b	MS-based screen	cell surface protein	PTPRC, PTPRJ
PLXNB2		MS-based screen	cell surface protein	SEMA4D, RAC2, PLXNB1
PLXND1		MS-based screen	cell surface protein	NRP1
PRNP	CD230	MS-based screen	cell surface protein	PRNP, NCAM1, HSPA5
PTPRM		MS-based screen	cell surface protein	PTPRC (CD45)
PVRL2	CD112	MS-based screen	cell surface protein	PVRL1,TNFRSF 14
PVRL3	CD113	MS-based screen	cell surface protein	PVRL1,2
RAC1		GEP analysis	cell surface protein	
RECK		MS-based screen	cell surface protein	MMP9, MMP14,TIMP1
SLC3A2	CD98	MS-based screen	cell surface protein	
TGFBR2		GEP analysis	cell surface protein	TGFB1, ENG

(*Continued*)

Entrez gene symbol	CD annotation	Screen selected criteria	Biological property	Interacting proteins on ALL
THY1	CD90	MS-based screen	cell surface protein	ITGAM, ITGB2
TPBG		MS-based screen	cell surface protein	HLA-C
TSPAN4		MS-based screen	cell surface protein	ITGB1, CD81
VASN		MS-based screen	cell surface protein	TGFB1
VCAM1	CD106	MS-based screen	cell surface protein	ITGB1, ITGB7, ITGA9
ZYX		MS-based screen	cell surface protein	ITGB1
IL1R1	CD121a	GEP analysis	cytokines expressed by MSCs	
IL-3		GEP analysis	cytokines expressed by MSCs	
IL-6		GEP analysis	cytokines expressed by MSCs	
IL-7		GEP analysis	cytokines expressed by MSCs	
KITLG		GEP analysis	cytokines expressed by MSCs	
SDF-1		GEP analysis	cytokines expressed by MSCs	
TSLP		GEP analysis	cytokines expressed by MSCs	
CD99	CD99	GEP analysis	Growth factors expressed by MSCs	
ENG	CD105	GEP analysis	Growth factors expressed by MSCs	
FGF2		GEP analysis	Growth factors expressed by MSCs	
FGF7		GEP analysis	Growth factors expressed by MSCs	
FGFR1		GEP analysis	Growth factors expressed by MSCs	
VEGFA		GEP analysis	Growth factors expressed by MSCs	
VEGFC		GEP analysis	Growth factors expressed by MSCs	
JAG1	CD339	GEP analysis	notch signaling	
JAG2		GEP analysis	notch signaling	
MAML1		GEP analysis	notch signaling	
MAML2		GEP analysis	notch signaling	
MAML3		GEP analysis	notch signaling	

(*Continued*)

Entrez gene symbol	CD annotation	Screen selected criteria	Biological property	Interacting proteins on ALL
Notch1		GEP analysis	notch signaling	
Notch3		GEP analysis	notch signaling	
Notch4		GEP analysis	notch signaling	
ACVR1		GEP analysis	TGF beta signaling	
ACVR2A		GEP analysis	TGF beta signaling	
BMP1		GEP analysis	TGF beta signaling	
BMP4		GEP analysis	TGF beta signaling	
SMAD4		GEP analysis	TGF beta signaling	
TGFB1		GEP analysis	TGF beta signaling	
TGFB2		GEP analysis	TGF beta signaling	
TGFBR1		GEP analysis	TGF beta signaling	
ALPL		MS-based screen	other	LNPEP
CTSB		MS-based screen	other	CTSA, CTSB
FAT1		MS-based screen	other	
HSPG2		MS-based screen	other	DAG1
NF2		MS-based screen	other	
NEGR1		MS-based screen	other	NEGR1
Notch 2		MS-based screen	other	
NPTN		MS-based screen	other	
RDX		MS-based screen	other	
THBS1		MS-based screen	other	CD47
VIM		MS-based screen	other	

Supplementary Table S3. siRNA screen-110 genes

			VHR-01			
	Genes	exp1	exp2	exp3	average	SD
1	ACVR1	122.45	104.66	111.02	112.71	9.02
2	ACVR2	101.62	107.12	70.73	93.16	19.61
3	ADAM15	101.62	78.63	92.41	90.89	11.57
4	ADAM17	101.80	95.25	102.91	99.99	4.14
5	ADAM9	73.30	84.96	124.71	94.33	26.95
6	ALCAM	105.45	109.31	115.49	110.08	5.07
7	ALPL	116.49	83.10	61.75	87.11	27.59
8	ANPEP	84.76	121.65	112.25	106.22	19.17
9	ANTXR1	104.53	110.20	82.51	99.08	14.62
10	ASAM	113.72	93.30	102.54	103.18	10.23
11	BMP1	105.57	103.89	52.62	87.36	30.09
12	BMP4	101.39	72.30	66.86	80.18	18.56
13	CD109	138.38	96.23	109.19	114.6	21.59
14	CD151	146.47	114.53	129.54	130.18	15.98
15	CD164L1	101.57	133.15	111.32	115.34	16.17
16	CD276	80.99	119.13	85.13	95.09	20.93
17	CD44	64.90	85.29	115.35	88.51	25.38
18	CD59	70.39	108.80	97.63	92.28	19.76
19	CD63	85.31	118.25	78.34	93.97	21.32
20	CD99	108.13	104.93	70.42	94.49	20.91
21	CDC42BPA	80.14	100.84	129.80	103.59	24.94
22	CDC42BPB	73.21	104.61	135.90	104.57	31.34
23	CDGAP	102.96	106.05	132.46	113.82	16.22
24	CDH2	86.33	90.18	101.12	92.54	7.67
25	CTNNB1	78.38	65.88	87.93	77.40	11.06
26	CTSB	82.17	81.98	93.84	86.00	6.79
27	CXC12L	84.62	103.86	92.97	93.82	9.65
28	DAF	80.74	81.01	113.61	91.78	18.90
29	DKK1	99.63	86.82	85.67	90.71	7.75
30	DKK2	125.31	85.34	131.11	113.92	24.92
31	EGFR	99.03	108.10	47.05	84.73	32.94
32	EMP3	93.67	99.91	89.36	94.31	5.30
33	ENG	98.01	87.62	120.53	102.05	16.82
34	ERBB2	127.67	114.39	119.64	120.56	6.69
35	FAS	75.15	100.47	101.58	92.40	14.95

(Continued)

	Genes	exp1	exp2	exp3	average	SD
36	FAT	103.60	110.38	92.28	102.09	9.14
37	FGF2	65.06	97.81	100.37	87.75	19.69
38	FGF7	50.76	93.20	104.94	82.97	28.50
39	FGFR1	121.66	81.52	91.70	98.29	20.87
40	FN1	107.81	92.13	71.59	90.51	18.16
41	FZD6	67.48	93.02	85.11	81.87	13.07
42	FZD7	72.15	79.45	117.94	89.85	24.60
43	HSPG2	125.77	116.71	136.66	126.38	9.99
44	ICAM1	125.03	104.24	156.07	128.45	26.09
45	IGF2R	95.43	80.96	127.02	101.14	23.55
46	IL1R1	64.90	70.67	77.52	71.03	6.32
47	IL3	62.36	78.26	94.64	78.42	16.14
48	IL6	87.34	79.84	115.44	94.21	18.77
49	IL7	57.76	88.76	85.87	77.46	17.13
50	ITGA11	101.25	111.82	71.75	94.94	20.77
51	ITGA3	103.83	106.01	77.06	95.63	16.12
52	ITGA5	101.48	105.59	129.91	112.32	15.36
53	ITGAV	80.60	118.95	118.08	105.88	21.89
54	ITGB1	91.69	99.81	130.60	107.37	20.53
55	ITGB5	73.72	97.16	111.47	94.12	19.06
56	JAG1	91.36	94.69	82.76	89.61	6.16
57	JAG2	55.94	95.86	83.85	78.55	20.48
58	KITLG	88.27	87.66	100.79	92.24	7.41
59	LAMP1	76.35	75.09	83.85	78.43	4.74
60	LEPR	128.22	114.76	80.85	107.94	24.41
61	LO11C4990	107.25	134.45	103.32	115.01	16.95
62	LRP1	120.69	89.99	78.14	96.27	21.96
63	MAML1	84.02	105.17	131.84	107.01	23.96
64	MAML2	88.64	120.39	111.29	106.77	16.35
65	MAML3	87.02	110.24	128.95	108.74	21.01
66	MSN	89.79	66.71	115.16	90.55	24.23
67	NEGR1	86.37	77.98	63.23	75.86	11.72
68	NF2	98.38	93.02	98.14	96.51	3.03
69	NOTCH1	157.37	91.53	146.76	131.88	35.35
70	NOTCH2	87.71	106.05	102.83	98.86	9.79
71	NOTCH3	101.76	112.38	73.29	95.81	20.21
72	NOTCH4	74.36	88.97	78.53	80.62	7.52

(Continued)

	Genes	exp1	exp2	exp3	average	SD
73	NRP1	79.01	95.16	116.57	96.91	18.84
74	NT5E	85.13	103.21	115.25	101.20	15.16
75	P2RX4	69.12	116.81	109.29	98.41	25.64
76	PDGFRB	43.51	97.11	57.13	65.92	27.86
77	PLXNB2	72.89	70.53	84.16	75.86	7.29
78	PLXND1	63.60	78.40	103.00	81.67	19.90
79	PRNP	69.93	63.97	73.22	69.04	4.69
80	PTPRM	72.75	125.65	93.38	97.26	26.66
81	PVRL2	114.18	105.31	70.22	96.57	23.25
82	PVRL3	75.98	66.85	109.02	83.95	22.19
83	RAC1	57.83	95.34	102.73	85.30	24.08
84	RAC2	68.68	122.91	93.64	95.08	27.14
85	RAC3	72.15	92.46	84.15	82.92	10.21
86	RDX	67.85	83.52	55.05	68.81	14.26
87	RECK	70.25	106.98	76.14	84.46	19.73
88	RHOA	71.18	68.95	110.83	83.65	23.56
89	RHOB	80.65	111.69	74.72	89.02	19.85
90	RHOC	96.77	89.66	74.35	86.93	11.46
91	RHOG	114.13	86.86	59.34	86.78	27.40
92	RHOQ	65.54	106.01	68.37	79.97	22.59
93	SDFR1/NPTN	57.92	68.06	76.64	67.54	9.37
94	SLC3A2	102.91	65.81	85.51	84.74	18.56
95	SMAD4	87.39	82.77	90.16	86.77	3.73
96	TGFB1	89.10	85.10	98.99	91.06	7.15
97	TGFB2	61.15	83.89	80.43	75.16	12.25
98	TGFBR1	159.77	89.80	74.89	108.15	45.32
99	TGFBR2	114.73	104.84	72.60	97.39	22.03
100	THBS1	35.52	92.18	82.72	70.14	30.35
101	THY1	132.42	137.85	75.71	115.33	34.42
102	TM4SF7	91.09	103.49	76.64	90.41	13.44
103	TPBG	51.92	83.43	75.81	70.39	16.44
104	TSLP	68.13	111.55	59.20	79.62	28.00
105	VCAM1	68.94	87.42	86.63	81.00	10.45
106	VEGF	79.31	83.10	86.13	82.85	3.42
107	VEGFC	77.92	78.58	60.18	72.23	10.44
108	VIM	98.80	85.01	65.94	83.25	16.50
109	WISP1	54.13	80.07	80.37	71.52	15.06
110	ZYX	68.73	90.50	115.18	91.47	23.24

(*Continued*)

VHR-03						
	Genes	exp1	exp2	exp3	average	SD
1	ACVR1	166.05	76.02	136.35	126.14	45.87
2	ACVR2	69.99	85.51	91.18	82.22	10.97
3	ADAM15	35.63	12.92	61.12	36.56	24.11
4	ADAM17	148.47	91.23	121.53	120.41	28.64
5	ADAM9	142.76	74.27	76.03	97.68	39.04
6	ALCAM	121.95	74.57	95.06	97.19	23.76
7	ALPL	87.22	73.01	55.14	71.79	16.07
8	ANPEP	137.91	93.25	103.23	111.46	23.44
9	ANTXR1	87.10	71.78	63.53	74.14	11.96
10	ASAM	42.65	58.63	65.88	55.72	11.89
11	BMP1	108.78	39.31	72.08	73.39	34.76
12	BMP4	86.71	56.23	56.94	66.63	17.40
13	CD109	149.77	80.29	95.35	108.47	36.55
14	CD151	139.33	145.75	142.96	142.68	3.22
15	CD164L1	92.50	97.33	80.51	90.11	8.66
16	CD276	95.30	88.18	94.31	92.60	3.86
17	CD44	101.37	99.77	87.19	96.11	7.77
18	CD59	63.74	55.70	73.06	64.16	8.69
19	CD63	110.95	69.16	61.15	80.42	26.74
20	CD99	189.26	88.14	118.46	131.95	51.89
21	CDC42BPA	85.57	128.67	62.84	92.36	33.44
22	CDC42BPB	203.57	126.61	136.61	155.60	41.85
23	CDGAP	132.23	45.75	80.08	86.02	43.55
24	CDH2	100.62	24.32	68.15	64.36	38.29
25	CTNNB1	83.43	51.42	61.09	65.31	16.42
26	CTSB	91.01	54.44	68.36	71.27	18.46
27	CXC12L	108.62	85.44	72.81	88.96	18.17
28	DAF	75.71	83.11	71.50	76.77	5.88
29	DKK1	73.47	87.48	52.52	71.15	17.59
30	DKK2	54.00	101.83	56.47	70.77	26.93
31	EGFR	73.15	65.54	82.36	73.68	8.43
32	EMP3	43.10	70.87	58.05	57.34	13.90
33	ENG	99.32	61.39	64.99	75.23	20.94
34	ERBB2	93.09	116.62	68.81	92.84	23.91
35	FAS	78.55	62.52	54.53	65.20	12.23
36	FAT	78.39	94.17	89.28	87.28	8.07

(*Continued*)

	Genes	exp1	exp2	exp3	average	SD
37	FGF2	97.71	133.82	67.05	99.52	33.42
38	FGF7	109.21	81.93	72.66	87.93	19.00
39	FGFR1	123.29	68.05	92.30	94.55	27.69
40	FN1	47.09	38.59	66.83	50.84	14.48
41	FZD6	38.11	56.21	69.98	54.77	15.98
42	FZD7	48.96	74.00	51.78	58.25	13.72
43	HSPG2	128.96	137.32	79.82	115.37	31.07
44	ICAM1	107.48	141.94	78.33	109.25	31.84
45	IGF2R	102.59	96.61	77.58	92.26	13.06
46	IL1R1	67.08	80.79	64.35	70.74	8.81
47	IL3	98.49	125.20	76.06	99.92	24.60
48	IL6	93.57	137.36	64.13	98.35	36.85
49	IL7	79.33	93.56	38.16	70.35	28.77
50	ITGA11	79.06	130.92	64.12	91.37	35.06
51	ITGA3	45.56	124.13	33.72	67.81	49.14
52	ITGA5	39.69	129.78	32.16	67.21	54.32
53	ITGAV	97.19	101.83	61.84	86.96	21.87
54	ITGB1	91.12	175.10	62.47	109.57	58.54
55	ITGB5	44.50	124.48	36.89	68.62	48.52
56	JAG1	43.10	136.94	58.21	79.42	50.39
57	JAG2	90.49	121.73	56.78	89.67	32.48
58	KITLG	121.95	141.36	80.50	114.6	31.09
59	LAMP1	81.86	90.51	73.90	82.09	8.31
60	LEPR	110.55	30.46	75.95	72.32	40.17
61	LO11C4990	96.29	72.63	57.28	75.40	19.65
62	LRP1	45.46	70.68	57.86	58.00	12.61
63	MAML1	132.35	128.97	85.58	115.63	26.08
64	MAML2	164.71	106.10	116.15	128.99	31.34
65	MAML3	105.75	114.64	74.88	98.42	20.87
66	MSN	89.88	114.83	60.88	88.53	27.00
67	NEGR1	63.26	118.41	60.43	80.70	32.69
68	NF2	115.48	64.24	69.25	82.99	28.25
69	NOTCH1	132.39	56.35	81.24	89.99	38.77
70	NOTCH2	73.15	89.86	53.57	72.19	18.17
71	NOTCH3	65.54	74.95	46.23	62.24	14.64
72	NOTCH4	33.05	70.75	54.23	52.67	18.90
73	NRP1	99.91	73.85	80.51	84.76	13.54

(Continued)

	Genes	exp1	exp2	exp3	average	SD
74	NT5E	82.18	91.27	61.00	78.15	15.53
75	P2RX4	63.10	89.55	75.08	75.91	13.25
76	PDGFRB	52.67	82.58	78.4	71.22	16.19
77	PLXNB2	81.98	72.52	64.43	72.98	8.78
78	PLXND1	101.33	44.87	71.61	72.61	28.24
79	PRNP	107.60	44.53	95.53	82.55	33.48
80	PTPRM	64.32	60.98	79.72	68.34	9.99
81	PVRL2	68.22	70.07	54.91	64.4	8.27
82	PVRL3	69.84	58.19	46.17	58.07	11.83
83	RAC1	43.75	92.64	89.50	75.3	27.37
84	RAC2	66.25	117.69	59.05	81.00	31.98
85	RAC3	91.48	97.98	96.48	95.31	3.40
86	RDX	75.67	99.39	92.45	89.17	12.19
87	RECK	68.86	133.78	51.63	84.75	43.32
88	RHOA	77.88	133.66	55.89	89.14	40.09
89	RHOB	66.73	65.61	85.14	72.49	10.96
90	RHOC	70.08	64.22	93.17	75.82	15.31
91	RHOG	84.60	54.78	84.57	74.65	17.21
92	RHOQ	67.75	63.36	72.22	67.78	4.43
93	SDFR1/NPTN	81.99	110.26	43.88	78.71	33.31
94	SLC3A2	43.15	75.69	50.23	56.36	17.11
95	SMAD4	97.08	44.13	75.43	72.21	26.62
96	TGFB1	114.38	52.80	87.62	84.93	30.88
97	TGFB2	87.81	43.92	67.61	66.45	21.97
98	TGFBR1	24.50	38.70	68.99	44.06	22.72
99	TGFBR2	107.13	102.21	85.85	98.40	11.14
100	THBS1	81.98	46.88	60.26	63.04	17.71
101	THY1	72.48	58.56	87.13	72.72	14.29
102	TM4SF7	53.21	72.61	77.03	67.62	12.67
103	TPBG	39.13	49.6	62.56	50.43	11.74
104	TSLP	54.11	48.08	63.51	55.23	7.77
105	VCAM1	53.33	75.49	60.57	63.13	11.30
106	VEGF	57.74	64.75	83.30	68.60	13.21
107	VEGFC	44.71	55.10	27.73	42.51	13.82
108	VIM	91.37	63.76	74.53	76.56	13.92
109	WISP1	63.61	72.38	51.88	62.62	10.29
110	ZYX	53.80	85.23	93.15	77.39	20.81

(*Continued*)

VHR-04						
	Genes	exp1	exp2	exp3	average	SD
1	ACVR1	156.92	185.84	143.21	161.99	21.77
2	ACVR2	85.64	87.85	118.04	97.17	18.10
3	ADAM15	85.77	90.04	117.41	97.74	17.17
4	ADAM17	134.37	125.93	94.10	118.13	21.24
5	ADAM9	108.95	79.03	116.79	101.59	19.93
6	ALCAM	108.33	103.19	142.19	117.91	21.19
7	ALPL	81.35	120.21	112.31	104.62	20.54
8	ANPEP	108.10	71.35	122.43	100.63	26.34
9	ANTXR1	97.49	72.12	110.76	93.45	19.63
10	ASAM	93.32	137.13	106.31	112.26	22.50
11	BMP1	117.45	131.12	128.07	125.55	7.18
12	BMP4	93.58	131.32	135.32	120.07	23.03
13	CD109	132.69	114.2	72.39	106.43	30.89
14	CD151	135.03	129.41	89.02	117.82	25.10
15	CD164L1	111.33	114.49	78.01	101.27	20.21
16	CD276	107.19	113.92	114.28	111.80	3.99
17	CD44	122.10	78.74	58.30	86.38	32.58
18	CD59	122.27	117.06	141.32	126.89	12.77
19	CD63	115.59	130.98	139.94	128.84	12.32
20	CD99	95.34	149.48	130.85	125.22	27.50
21	CDC42BPA	87.34	100.14	115.62	101.04	14.16
22	CDC42BPB	47.22	62.19	79.17	62.86	15.98
23	CDGAP	107.97	114.87	47.56	90.13	37.03
24	CDH2	80.13	92.72	59.29	77.38	16.88
25	CTNNB1	82.87	101.81	73.56	86.08	14.40
26	CTSB	82.84	90.12	71.35	81.44	9.47
27	CXC12L	103.61	91.66	99.05	98.11	6.03
28	DAF	121.13	61.82	56.96	79.97	35.73
29	DKK1	87.33	57.82	109.98	85.04	26.16
30	DKK2	111.23	134.8	101.29	115.78	17.21
31	EGFR	111.49	124.93	123.95	120.12	7.49
32	EMP3	88.76	117.59	90.45	98.94	16.18
33	ENG	99.57	94.52	87.26	93.78	6.19
34	ERBB2	123.11	111.34	71.83	102.09	26.87
35	FAS	55.61	70.02	43.91	56.52	13.08
36	FAT	70.85	124.59	108.37	101.27	27.56

(Continued)

	Genes	exp1	exp2	exp3	average	SD
37	FGF2	119.40	122.16	34.12	91.90	50.05
38	FGF7	97.00	118.59	101.92	105.84	11.32
39	FGFR1	111.36	98.24	116.61	108.73	9.46
40	FN1	76.39	129.89	63.14	89.80	35.34
41	FZD6	81.92	68.62	53.59	68.04	14.18
42	FZD7	56.98	95.28	41.14	64.47	27.84
43	HSPG2	71.65	124.21	46.84	80.90	39.51
44	ICAM1	107.06	148.38	68.51	107.98	39.94
45	IGF2R	95.08	77.96	41.14	71.39	27.56
46	IL1R1	79.71	78.32	65.74	74.59	7.70
47	IL3	116.41	133.98	82.13	110.84	26.37
48	IL6	106.45	111.44	108.01	108.63	2.55
49	IL7	87.39	74.59	112.34	91.44	19.20
50	ITGA11	129.14	164.01	147.32	146.83	17.44
51	ITGA3	133.15	112.2	41.56	95.63	47.99
52	ITGA5	82.05	112.58	48.99	81.21	31.81
53	ITGAV	85.05	89.42	64.12	79.53	13.52
54	ITGB1	94.10	123.69	86.07	101.29	19.81
55	ITGB5	81.31	95.19	76.75	84.42	9.60
56	JAG1	68.35	101.67	123.68	97.90	27.86
57	JAG2	113.22	112.35	73.35	99.64	22.77
58	KITLG	140.27	114.44	67.80	107.5	36.73
59	LAMP1	64.73	99.81	43.71	69.42	28.34
60	LEPR	98.82	128.55	104.69	110.69	15.74
61	LO11C4990	90.36	78.07	85.08	84.50	6.16
62	LRP1	82.35	103.19	104.96	96.83	12.58
63	MAML1	111.59	120.02	145.18	125.59	17.47
64	MAML2	112.24	104.91	102.37	106.50	5.13
65	MAML3	115.72	125.02	72.63	104.46	27.95
66	MSN	71.02	104.39	43.26	72.89	30.61
67	NEGR1	90.00	89.18	49.17	76.12	23.34
68	NF2	108.85	134.80	61.26	101.64	37.30
69	NOTCH1	107.16	75.12	64.12	82.13	22.36
70	NOTCH2	163.82	94.04	125.11	127.66	34.96
71	NOTCH3	126.99	124.26	75.68	108.98	28.87
72	NOTCH4	73.25	92.09	49.35	71.56	21.42
73	NRP1	90.10	98.81	69.32	86.07	15.15

(*Continued*)

	Genes	exp1	exp2	exp3	average	SD
74	NT5E	120.61	118.21	48.27	95.70	41.09
75	P2RX4	95.40	85.89	40.30	73.87	29.45
76	PDGFRB	78.44	80.51	66.01	74.98	7.84
77	PLXNB2	98.56	56.29	63.23	72.69	22.67
78	PLXND1	97.94	75.21	67.62	80.26	15.78
79	PRNP	128.06	67.64	120.64	105.45	32.95
80	PTPRM	96.64	164.73	171.15	144.17	41.29
81	PVRL2	110.58	139.28	74.24	108.03	32.59
82	PVRL3	106.93	107.20	76.84	96.99	17.45
83	RAC1	111.88	78.36	81.86	90.70	18.43
84	RAC2	112.76	106.34	54.81	91.30	31.77
85	RAC3	64.33	80.22	44.72	63.09	17.78
86	RDX	102.11	85.65	55.44	81.07	23.67
87	RECK	108.17	74.30	83.29	88.59	17.54
88	RHOA	151.31	62.58	106.13	106.67	44.37
89	RHOB	78.44	58.58	134.52	90.51	39.38
90	RHOC	100.13	98.14	156.37	118.21	33.06
91	RHOG	111.33	147.71	130.13	129.72	18.20
92	RHOQ	79.78	137.7	86.51	101.33	31.68
93	SDFR1/NPTN	71.39	76.07	69.20	72.22	3.51
94	SLC3A2	43.50	72.81	74.42	63.58	17.41
95	SMAD4	91.11	96.47	56.87	81.48	21.48
96	TGFB1	102.63	90.75	94.31	95.90	6.10
97	TGFB2	75.51	86.70	75.95	79.39	6.34
98	TGFBR1	74.37	189.47	109.35	124.4	59.00
99	TGFBR2	89.15	126.88	100.49	105.51	19.36
100	THBS1	73.95	133.65	65.92	91.17	37.01
101	THY1	73.39	239.28	204.55	172.41	87.49
102	TM4SF7	102.18	128.46	79.26	103.3	24.62
103	TPBG	87.33	117.25	93.86	99.48	15.73
104	TSLP	59.55	124.83	63.95	82.78	36.49
105	VCAM1	81.01	82.46	50.87	71.45	17.84
106	VEGF	90.27	86.56	65.11	80.65	13.58
107	VEGFC	98.99	83.08	88.57	90.22	8.08
108	VIM	76.81	143.99	81.14	100.65	37.60
109	WISP1	62.58	123.16	96.54	94.10	30.37
110	ZYX	103.9	226.17	147.5	159.19	61.96
